# Review of tuberculosis treatment outcome reporting system in Denmark, a retrospective study cohort study from 2009 through 2014

**DOI:** 10.1186/s12913-020-4927-y

**Published:** 2020-02-03

**Authors:** Inge K. Holden, Peter H. Andersen, Christian Wejse, Troels Lillebaek, Isik S. Johansen

**Affiliations:** 1 Department of Infectious Diseases, Odense University Hospital, Odense, Denmark; 2 Department of Clinical Research, University of Southern Denmark, Odense, Denmark; 3 Mycobacterial Centre for Research Southern Denmark – MyCRESD, Odense, Denmark; 4 Department of Infectious Disease Epidemiology and Prevention, Statens Serum Institut, Copenhagen, Denmark; 5 Department of Infectious Diseases, Aarhus University Hospital, Aarhus, Denmark; 6 GloHAU, Center for Global Health, Department of Public Health, Aarhus University, Aarhus, Denmark; 7 International Reference Laboratory of Mycobacteriology, Statens Serum Institut, Copenhagen, Denmark; 8 Global Health Section, Department of Public Health, University of Copenhagen, Copenhagen, Denmark

**Keywords:** Tuberculosis treatment outcome, Lost to follow-up, Surveillance, Review of tuberculosis treatment outcome

## Abstract

**Background:**

In Denmark, reporting of tuberculosis (TB) treatment outcome is voluntary and data incomplete. In the European Centre for Disease Prevention and Control most recent report presenting data from 2017, only 53.9% of Danish pulmonary TB cases had a reported outcome. Monitoring of TB treatment outcome is not feasible based on such limited results. In this retrospective study from 2009 to 2014, we present complete treatment outcome data and describe characteristics of cases lost to follow up.

**Methods:**

All cases notified from 2009 through 2014 were reviewed. Hospital records were examined, and TB treatment outcome was categorized according to the World Health Organization’s (WHO) definitions.

**Results:**

A total of 2131 TB cases were included. Treatment outcome was reported to the Surveillance Unit in 1803 (84.6%) cases, of which 468 (26.0%) were reclassified. For pulmonary TB, 339 (28.9%) cases were reclassified between cured and treatment completed.

Overall, the proportion of cases who achieved successful treatment outcome increased from 1488 (70.4%) to 1748 (81.8%).

**Conclusion:**

A high number of cases were reclassified during the review process. Increased focus on correct treatment outcome reporting is necessary in Denmark. A more comprehensive and exhaustive categorization of TB treatment outcome could be beneficial, especially for cases where collection of sputum or tissue towards the end of treatment is challenging.

## Background

Successful treatment of infectious tuberculosis (TB) cases is key to TB control. Therefore, surveillance of TB treatment outcome is fundamental when evaluating TB programs.

In Denmark, standardized voluntary reporting of TB treatment outcome was initiated in 2000. Since then, results have been reported to the Danish national TB surveillance unit at the Department of Infectious Disease Epidemiology & Prevention at Statens Serum Institut (SSI). Because reporting TB treatment outcome is voluntary, data are incomplete and delayed. E.g. in the most resent rapport from European Centre for Disease Prevention and Control (ECDC), only 53.9% of notified Danish TB cases had information on treatment outcome [1]. Thus, timely TB treatment outcome monitoring it is not possible based on such incomplete results. Furthermore, previous studies have suggested that a more comprehensive and exhaustive categorization of TB treatment outcome might be useful, as the present categorization does not take into account TB patients who require prolonged treatment, who die of other causes than TB or difficulties related to tissue and sputum sampling near the end of TB treatment [2, 3].

In addition, in Denmark, it has not been evaluated what happens to patients reported as lost to follow up (LTFU). These patients are at personal risk and represent a public health risk. There is no information on their whereabouts, there is a potential risk of transmission, they may develop drug resistance and more severe disease with complications.

The aims of this study were to evaluate the TB treatment outcome system in Denmark and report more complete data from 2009 through 2014. Additionally, we aimed to describe what happens to patients LTFU.

## Methods

From 2009 through 2014, all patients notified with TB in Denmark were included as described in detail earlier [4]. In brief, notification data was obtained from the Danish national TB surveillance unit which also provided Civil Registration Number – CRN, date of notification and reported treatment outcome. Microbiologic data was provided by the International Reference Laboratory of Mycobacteriology at SSI. Finally, all hospital records were reviewed for socio-demographics, clinical characteristics, TB treatment, and treatment outcome. Treatment outcome is reported to the Danish national TB surveillance unit in accordance with the WHO’s definitions. After reviewing the medical reports and microbiologic data, treatment outcome was (re)classified according to WHO’s definitions (Table 1) [5].

**Table 1:** Tuberculosis treatment outcome categories modified from WHO definitions [5]

Treatment outcome	Definition
Cured	TB confirmed by culture at the beginning of treatment and culture negative in the last month of treatment and on at least one previous occasion.
Treatment Completed	TB treatment completed without evidence of failure, but without fulfilling the above mention criteria
Treatment success	The sum of cured and treatment completed
Died	A TB patient who dies for any reason before starting or during TB treatment.
Treatment failed	Positive culture during last month of the continuation phase
Lost to follow-up	A TB patient who did not start treatment or whose treatment was interrupted for 2 consecutive months or more.
Transfer	A TB patient who permanently leaves Denmark during TB treatment
Not evaluated	A TB patient who does not fit into other categories
Still on treatment	A TB patient who were still on treatment at time of study termination

All data are individual and were cross-linked using the unique CRN, which is assigned to all residents of Denmark at time of birth or after residing legally in Denmark for 3 months. The CRN enables to follow patients across hospitals in Denmark and determine if the patient emigrated or died during the study period

### Statistical analyses

Categorical data was described by total and percentages, the denominator for calculated percentages was the number of cases with known information. Data comparisons were made using the chi-square test or Fisher’s exact test if 20% of expected cell value were ≤ 5. Continuous variables were described as medians and interquartile ranges and compared using the Wilcoxon rank sum test. A *p*-value of less than 0.05 (5%) was considered statistically significant. Logistic regression was used to investigate LTFU, risk factors were defined at baseline, Univariable and multivariable analyses between LTFU and potential risk factors were performed. The multivariable model was built in a forward method, variables for multivariate analysis were selected if they showed a univariate association with LTFU (*p* < .05) and included in the final model if this led to significant improvement.

## Results

**Fig. 1:**
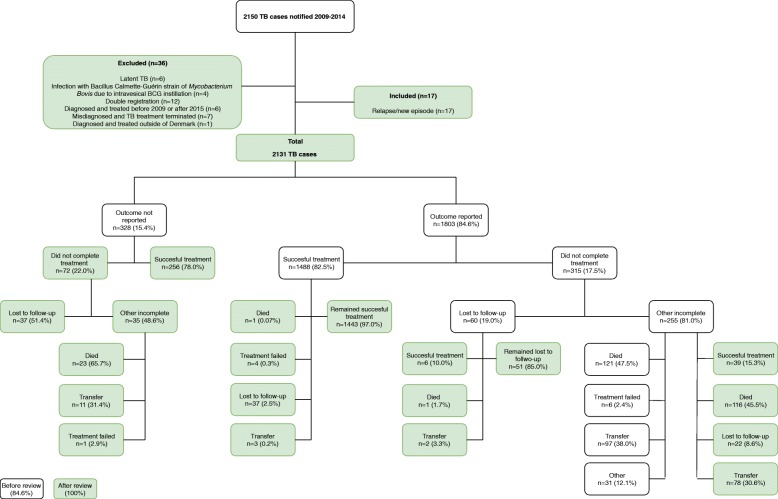
Review of TB treatment outcome, Denmark 2009–2014

Figure 1 illustrates the study population before and after review. From 2009 through 2014, a total of 2150 patients were notified with TB. During review, 36 cases were excluded, and 17 cases were included, resulting in a total of 2131 TB cases. Treatment outcome was reported to the Surveillance Unit in 1803 (84.6%) cases of which 468 cases (26.0%) were reclassified; 441 (94.2%) of these were diagnosed with PTB

After the review, cases classified as cured increased from 522 (24.7%) to 621 (29.1%) (Table 2). The proportion of cases who achieved successful treatment increased from 1488 (70.4%) to 1748 (81.8%) (Table 2). During the study period, successful treatment increased from 80.2% in 2009 to 85.5% in 2014 (*p* = 0.08).

**Table 2:** Tuberculosis treatment outcome; Denmark 2009–2014

	Before audit	After audit	Additional cases^a^	Total after audit
	n (%)	n (%)	n (%)	n (%)
Cured	522 (24.7)	619 (29.2)	2 (11.7)	621 (29.1)
Treatment completed	966 (45.7)	1114 (52.7)	8 (47.1)	1123 (52.7)
Successful treatment^b^	1488 (70.4)	1737 (81.9)	10 (58.8)	1748 (81.8)
Died	121 (5.7)	138 (6.5)	3 (17.7)	141 (6.6)
Treatment failed	6 (0.3)	4 (0.1)	1 (5.9)	5 (0.2)
Treatment interrupted	60 (2.8)	145 (6.9)	2 (11.7)	147 (6.9)
Transfer	97 (4.6)	93 (4.4)	1 (5.9)	94 (4.4)
Other	31 (1.5)	0 (0)	0 (0)	0 (0)
Missing	311 (14.7)	0 (0)	0 (0)	0 (0)
Total	2114	2114	17	2131

^a^Additional TB cases were included if a relapse/new episode of TB was identified in the patient record. A new episode/relapse was defined according to WHO/ECDC guidelines and cases were only included once during a 12 months period

^b^The sum of cured and treatment completed

In PTB cases, reclassification between cured and treatment completed occurred for 339 (28.9%) cases. Culture positive pulmonary cases accounted for 1425 (84.8%) of the PTB cases, of these, 621 (43.6%) were cured whereas 506 (35.5%) were categorized as completed.

In the category “died”, six patients were reclassified according to the WHO definition (Table 1) [5]. Four patients completed treatment before dying 2–8 months later, and one patient was LTFU six months prior to death. In addition, one patient was reported as treatment completed. This patient was prescribed 12 months of TB treatment due to poor adherence and died during the 10th month of treatment.

During the study period, additionally 137 patients died after TB treatment outcome was reported. The median time from TB treatment termination to death was 1.7 years (IQR: 0.8–2.9). Eighteen (13.1%) patients had previously been LTFU, the remaining patients had completed TB treatment successfully.

A total of 9 patients in the TB treatment outcome category failure were reclassified; three patients from treatment completed to failure, 6 patients from failure to cured (*n* = 1) and to LTFU (*n* = 5) (Table 2).

For cases reported as not completing treatment (*n* = 315, 17.5%), LTFU accounted for 60 (19.0%) cases (Fig. 1). Of these, 51 cases (85.0%) remained LTFU, whereas 6 cases completed treatment, 2 patients returned to their native country, i.e. transferred out, one patient died during TB treatment and the last patient was categorized as treatment failure (Fig. 1)

After reviewing the remaining TB cases, another 96 cases were classified as LTFU (Fig. 1), resulting in a total of 147 (6.9%) cases (Table 2). The proportion of LTFU varied from 8.2% in 2010 to 5.7% in 2014

Among patients LTFU, 15 patients returned to the hospitals due to TB and re-initiated their TB treatment. Of these, 8 completed, 3 died during treatment and four were LTFU again. During the study period, three patients emigrated more than 3 years after they were LTFU. At the end of the study period 119 patients remained LTFU. The characteristics of all cases LTFU are presented in Table 3. Patients who were LTFU were significantly more frequent male, of Greenlandic origin, tobacco smokers, suffering from illegal drug abuse or homeless. The final multivariant model was fitted on 1813 cases. After adjusting for alcohol abuse and homelessness the association between Greenlandic origin and LTFU disappeared. There was a significantly greater risk of LTFU among male patients (*p* = 0.01) who suffered from alcohol abuse (*p* < 0.001) and homelessness (*p* < 0.001)

**Table 3:** Demographics and clinical characteristics after review

	LTFU (%)	Successful treatment^g^ (%)	*P*-value	OR (crude)	[95%CI^i^]	OR (adjusted)	[95%CI]
Total	147 (7.8)	1744 (92.2)					
Male	116 (78.9)	1030 (59.1)	< 0.001	2.59	1.69–3.98	2.2	1.41–3-45
Median age (IQR)	43 (28–49)	41 (29–53)	0.44				
Age: years			0.2				
0–24	23 (15.7)	276 (15.8)		1.46	0.82–7.86		
25–44	63 (42.9)	704 (40.4)		1.50	0.92–8.03		
45–64	56 (38.0)	612 (35.1)		1.54	0.94–8.26		
≥ 65	5 (3.4)	152 (8.7)		RF^h^			
Country of origin							
Danes	42 (28.6)	584 (33.5)	< 0.001	1.11	0.73–1.70	1.08	0.70–1.66
Greenlandic	47 (32.0)	262 (15.0)		2.78	1.82–4.24	1.90	1.16–3-13
Immigrants^b^	58 (39.4)	898 (51.5)		RF			
Predisposing factors:							
Alcohol abuse^a^	85 (61.2)	509 (30.4)	< 0.001	3.61	2.50–5.21		
Tobacco	116 (82.9)	900 (53.8)	< 0.001	4.16	2.61–6.63		
Cannabis	64 (48.5)	261 (16.1)	< 0.001	4.93	3.39–7.16		
Illegal drug use	26 (17.9)	111 (6.4)	< 0.001	3.19	1.93–5.29		
Homelessness	45 (30.8)	136 (7.8)	< 0.001	5.25	3.54–7.77	3.51	1.41–3-45
CCI = 0	99 (72.8)	1197 (70.0)	0.79	RF			
CCI = 1	19 (14.0)	262 (15.3)		0.88	0.53–1.46		
CCI ≥ 2	18 (13.2)	251 (14.7)		0.87	0.49–1.53		
Pulmonary TB^c^	126 (85.7)	1354 (77.6)	0.02	1.73	1.07–2.79		
Extrapulmonary TB^d^	21 (14.3)	390 (22.4)		RF			
Died after TB treatment termination	18 (12.2)	114 (6.5)	0.01				
Microscopy positive Pulmonary TB	37 (29.4)	356 (26.3)	0.46				
MDR^e^ TB	1 (0.8)	8 (0.6)					
XDR^f^ TB	0 (0)	1 (0.8)					
Treatment duration (months)	Median 4 (IQR 2–5)						

^a^Alcohol abuse was quantified according to the Danish Health Authorities recommendations (more than 14 units pr. week of alcohol for women and more than 21 units for men)

^b^Patients born abroad or those born in Denmark for whom one or both parents had been born abroad

^c^Any bacteriologically confirmed or clinically diagnosed case of TB involving the lungs, the tracheobronchial tree or the larynx including cases diagnosed with coexisting extrapulmonary TB

^d^Any bacteriologically confirmed or clinically diagnosed case of TB involving organs or anatomical sites other than the lungs, the tracheobronchial tree or the larynx

^e^Multidrug resistance tuberculosis

^f^Extensively drug-resistant tuberculosis

^g^The sum of cured and treatment completed

^h^Reference

^i^Confidence interval

## Discussion

This is the first study to evaluate the reporting of TB treatment outcome in Denmark. Overall, the review resulted in reclassification of one in four cases. The greatest proportion of reclassifications occurred between cured and treatment completed, mostly because the culture results were not available near end of treatment. Cases classified as treatment success and LTFU increased significantly during the review

In total, 15% of the patients did not have a reported treatment outcome. This figure is much lower than reported to ECDC for 2017 indicating a treatment outcome result for only 53.9% patients. The discrepancy is explained by delays in reporting TB treatment outcome data to national TB surveillance system in Denmark, as many treatment outcome results only are obtained after personal contact reminding the department/hospital to report

The fact that 29% PTB were reclassified from cured to treatment completed, and that 36% of culture positive PTB cases were classified as treatment completed, highlights the difficulties in achieving sputum samples at the end of treatment. Typically, the patients are asymptomatic towards the end of treatment and the importance in carefully instructing the patient in procedures to induce sputum sample might be neglected. Consequently, in numerous cases a sputum sample requires sputum induction, gastric lavage or bronchoscopy. These invasive procedures do involve potential risks and might not be applicable for all patients. This is even more evident for cases diagnosed with EPTB, where successful treatment outcome relies on clinical assessment supplemented with advanced imagining techniques, as repeated tissue sampling often requires invasive procedures. For these cases, it might be more beneficial to report treatment outcome one year after treatment completion where the majority of TB relapse have occurred [6, 7]. The relapse cases then should be categorized as treatment failure [3]. However, sputum/tissue sampling should still be prioritized in order to obtain bacteriologically confirmed cured in patients when possible

Our results also describe confusion between the outcomes “failure” and “interrupted”. All cases reported as treatment failures were reclassified as they did not have smear or culture positive sputum sample taken at month 5 or later during treatment. Five of the 6 cases were reclassified to LTFU

The review resulted in an increase in cases LTFU. The largest share was patients reclassified from successful treatment to LTFU (*n* = 37). These patients were typically LTFU during the last 2 months of TB treatment and might have been categorized as successful treatment as the clinician assumed the patient had completed treatment

Among patients who did not have a reported outcome, 11% were LTFU, these patients typically missed appointments repeatedly which eventually resulted in termination of their follow-up from the outpatient clinic. The protracted duration of outpatient course can potentially result in missed reporting of treatment outcome. Male patients who suffered from homelessness and alcohol abuse were at significantly greater risk of LTFU. This is not surprising as earlier studies have identified these risk factors to be associated with unsuccessful treatment [4, 8–11]. However, it does emphasize an increased focus on strategies maintaining this group in treatment is needed

At the end of the study period, 119 (5.6%) patients remained LTFU, the remaining patients had either died, transferred out or returned to hospital and completed TB treatment or died during treatment. The proportion of patient LTFU who died during the study period was significantly greater than patients who completed treatment successfully. Patients LTFU were not older or had more comorbidities. However, they the following risk factors were reported significantly more frequently; alcohol abuse, smoking, use of illegal drugs, and homelessness. These risk factors have been associated with mortality in earlier studies [4, 12–16]

An earlier study from United Kingdom reported a decrease in patients LTFU after review, however only patients recorded with the treatment outcome LTFU were reviewed. Hence, the factual number of LTFU was unknown, since the remaining notified cases were not reviewed [17]. We have similar results, as the number of cases recorded as LTFU decreased by 15%

Before review, six cases were classified as treatment failure. All these cases were reclassified, one of which had a nucleic acid amplification positive sputum sample which was culture and smear negative and reclassified to the outcome cured. The remaining cases were LTFU after 2–3 months of treatment

Patients who were classified as treatment failure after review had culture positive samples during the last month of treatment, resulting in extending the continuation phase. Consequently, the patients’ treatment outcome was reported at end of the extended TB treatment as cured/completed treatment

This is the first study to evaluate national TB treatment outcome during a 6-year period by systematic patient record review. This has only been possible due to having access to a national health registry that allows cross-reference with patients’ records (e.g. the Danish CRN system) has enabled this level of detailed study. However, this study has limitations. The number of cases reclassified might be underestimated, due to the voluntary reporting of TB treatment outcome; 15% of the total cases were not reported at all. Also, the study population was identified by notification data, consequently patients who were not notified could not be included. The underreporting of TB on the regional level was assessed to 7.5% in a recent Danish study, where the non-notified cases all were culture-negative and did not differ significantly in treatment outcome [18]. The number of patients who emigrated or died after TB treatment might be underestimated as 3.9% (*n* = 65) had temporary CRN and are therefore not registered in the CRN register. Finally, all clinical information was obtained from hospital records; hence no direct patient contact and the quantity and quality solely depends on the medical records. This can lead to information bias, risk factors such as alcohol and illegal drug use was missing in 5 and 8% of the cases and units of alcohol might be underreported

## Conclusion

A high number of cases were reclassified during review which emphasizes that increased focus on correct reporting of TB outcome is necessary in Denmark. A more comprehensive and exhaustive categorization of TB treatment outcome could be beneficial, especially in cases where sputum specimens or tissue sampling towards the end of treatment is challenging. Furthermore, we recommend reporting of TB treatment outcome to become mandatory in Denmark as an integrated part of notification for TB

## Data Availability

The datasets generated and analysed during the current study are available from the corresponding author on reasonable request

## Abbreviations

CI: Confidence interval
CRN: Civil registration number
ECDC: European Centre for Disease Prevention and Control
EPTB: Extrapulmonary tuberculosis
LTFU: Lost to follow-up
PTB: Pulmonary tuberculosis
RF: Reference
SSI: Statens Serum Institut
TB: Tuberculosis
WHO: World Health Organization
